# Clinical implications of thymidylate synthetase, dihydropyrimidine dehydrogenase and orotate phosphoribosyl transferase activity levels in colorectal carcinoma following radical resection and administration of adjuvant 5-FU chemotherapy

**DOI:** 10.1186/1471-2407-8-188

**Published:** 2008-07-02

**Authors:** Masashi Ishikawa, Takayuki Miyauchi, Yutaka Kashiwagi

**Affiliations:** 1 Department of Surgery, National Kochi Hospital, Kochi, Japan; 2 Department of Surgery, Tokushima Red Cross Hospital 103 Irinokuchi, Komatsushima-cho, Komatsushima City, Tokushima 773-8502, Japan

## Abstract

**Bckground:**

A number of studies have investigated whether the activity levels of enzymes involved in 5-fluorouracil (5-FU) metabolism are prognostic factors for survival in patients with colorectal carcinoma. Most reports have examined thymidylate synthetase (TS) and dihydropyrimidine dehydrogenase (DPD) in unresectable or metastatic cases, therefore it is unclear whether the activity of these enzymes is of prognostic value in colorectal cancer patients treated with radical resection and adjuvant chemotherapy with 5-FU.

**Methods:**

This study examined fresh frozen specimens of colorectal carcinoma from 40 patients who had undergone curative operation and were orally administered adjuvant tegafur/uracil (UFT) chemotherapy. TS, DPD and orotate phosphoribosyl transferase (OPRT) activities were assayed in cancer tissue and adjacent normal tissue and their association with clinicopathological variables was investigated. In addition, the relationships between TS, DPD and OPRT activities and patient survival were examined to determine whether any of these enzymes could be useful prognostic factors.

**Results:**

While there was no clear relationship between pathological findings and TS or DPD activity, OPRT activity was significantly lower in tumors with lymph node metastasis than in tumors lacking lymph node metastasis. Postoperative survival was significantly better in the groups with low TS activity and/or high OPRT activity.

**Conclusion:**

TS and OPRT activity levels in tumor tissue may be important prognostic factors for survival in Dukes' B and C colorectal carcinoma with radical resection and adjuvant chemotherapy with UFT.

## Background

Advanced colorectal carcinoma remains a significant health care problem in developed countries. Even with complete radical resection, almost half of patients develop local or distant recurrence presumably attributable to disseminated micro metastases present at the time of surgery. Administration of 5-fluorouracil (FU) significantly improves survival in patients with Dukes' C tumors and may also be beneficial in patients with Dukes' B tumors[1]. Nevertheless, a number of patients treated with adjuvant chemotherapy still experience tumor recurrence within 5 years. Identification of highly specific prognostic factors would aid in predicting the efficacy of anticancer agents before the initiation of such therapies.

Thymidylate synthetase (TS), dihydropyrimidine dehydrogenase (DPD) and orotate phosphoribosyl transferase (OPRT) are key enzymes in the regulation of 5-FU metabolism. TS is the rate-limiting enzyme in *de novo *pyrimidine biosynthesis, and is inhibited by the 5-FU metabolite 5-fluoro-2-deoxyuridylate (FdUMP), leading to inhibition of DNA synthesis. In clinical studies of various malignancies a correlation was observed between high levels of TS and 5-FU resistance[2,3]. DPD is the initial enzyme in the three-step metabolic pathway leading to the catabolism of the pyrimidine bases uracil and thymidine; 70–80% of administered 5-FU is degraded *in vivo *by DPD to fluorinated β-alanine. Several studies have demonstrated an association between high DPD levels and 5-FU resistance. In advanced cases of unresectable colorectal carcinoma, only those with low TS mRNA levels and low DPD levels were found to respond to 5-FU treatment[4,5]. Several *in vitro *studies suggested the major mechanism of 5-FU resistance to be a marked decrease in 5-FU phosphorylation by enzymes such as OPRT, which changes 5-FU to FUMP in the presence of 5-phosphoribosyl-1-pyrophosphate. At present, it is still unclear whether OPRT is a key activator of 5-FU and an accurate prognosticator of its anti-tumor effects in a clinical setting. Several studies have demonstrated that the activities of 5-FU metabolizing enzymes such as TS, DPD and OPRT predict the efficacy of chemotherapy with 5-FU for unresectable metastatic colorectal cancer[6,7]; however, few reports have investigated the relationship between the activity of these enzymes and duration survival in patients with colorectal carcinoma receiving radical operation combined with adjuvant oral 5-FU chemotherapy[8,9].

Here, we analyze the ability of TS, DPD and OPRT activity levels to predict the survival of colorectal carcinoma patients receiving radical operation and adjuvant tegafur/uracil, UFT^®^. It is not clear whether malignant processes contribute to changes in the activities of these enzymes within tumors; therefore, the present study also compared enzyme activity in cancer tissue and surrounding normal tissue.

## Methods

### Patients

Forty patients (23 males, 17 females; mean age of 68 ± 7 years) underwent their first colorectal resection at National Kochi Hospital between April 2000 and April 2002. The experimental protocol was approved by the Research Committee of National Kochi Hospital. All patients were informed of the nature and risk of this study, and written informed consent was obtained. The patients were divided into two groups according to Dukes' stage. Patients with Dukes' B corresponded with UICC stage II A (T3pN0, n = 15) and UICC stage III A (T4pN0, n = 7), and those with Dukes' C corresponded with UICC IIIA (T3pN1, n = 11), UICC stage III B (T4pN1, n = 6) and UICC stage III C (T4pN2, n = 1). Details concerning age, gender, site and size of tumor and pathological findings including histological classification, lymph node metastasis and depth of tumor invasion are presented in Table 1. All patients underwent radical operation with lymph node dissection and received 300 mg/day oral tegafur/uracil (UFT^®^; Taiho pharmaceutical Co., Japan), a fluoropyrimidine inhibitor of dihydropyrimidine dehydrogenase, containing tegafur and uracil in a 1:4 molar ratio) for a total of two years. No patient received radiation or chemotherapy before surgery or enteral nutrition or other chemotherapy during the study.

**Table 1:** Clinical and pathological parameters

	Age (yrs)	Male: Female	Histological Type Well:mod	Tumor Location Colon: rectum	Nodal Status N0:N1:N2	Invasion Depth ss:se	Tumor Size(cm)
Dukes'B	67.9 ± 10.7	13:9	12:10	14:8	22:0:0	15:7	5.3 ± 1.3
Dukes'C	68.8 ± 5.6	10:8	10:8	12:6	0:17:1	11:7	5.5 ± 1.3

*s*.*s*.: histological tumor invasion of subserosa, *s*.*e*.: histological tumor invasion of serosa. Values represent mean ± SD.

### Methods

We investigated the relationship between clinical pathophysiological characteristics and the activities of TS, DPD and OPRT in 40 patients. We also studied whether the activities of these enzymes were risk factors for recurrence.

Tissue was taken from the tumor and adjacent tumor-free sites (> 5 cm from tumor) of the resected sample, and immediately frozen in liquid nitrogen for preservation of enzymatic activity. Samples were stored at -80°C until use. TS, DPD and OPRT activities were examined in frozen specimens as described by Fujii et al [8] as follows.

### TS activity

TS enzyme activity was measured by tritium release assay. Tissue was homogenized in 50 nM Tris HCl (pH 7.3) containing 2 nM dithiothreitol. After ultracentrifugation (105000 × g, 1 h, 4°C), the supernatant was collected and incubated at 37°C with methylene tetrahydrofolic acid and the substrate, [^3^H]-dUMP. Aliquots of the reaction mixture were removed after 10, 20 and 30 min of incubation and the reaction was stopped immediately by adding 10% active carbon suspension containing 4% trichloroacetic acid. After centrifugation, ^3^H_2_O in the supernatant was quantified with a liquid scintillation counter. The reaction rate was obtained from the relationship between reaction time and the amount of ^3^H_2_O formed. From this reaction rate and the protein concentration (determined separately), TS activity (pmol/min per mg protein) was calculated.

### DPD activity

DPD enzyme activity was measured in a sample of the stored tissue by radioisotope-high performance liquid chromatography (RI-HPLC). The tissue sample was homogenized in 20 mM phosphate buffer (pH 8.0) containing 1 mM ethylenediamine tetraacetic acid (EDTA)·2 K and 1 nM 2-mercaptoethanol. After ultracentrifugation (105000 × g, 1 h, 4°C), the supernatant was collected and incubated at 37°C in the presence of 6.25 mM nicotinamide adenine dinucleotide phosphate (NADPH) and 125 M [^3^H]-5-FU (25 Ci/ml). Aliquots of the reaction mixture were removed after 10, 20, and 30 min of incubation, and the reaction was stopped immediately by adding an equal volume of 5% HClO_4_. The aliquots were diluted 1:2 with a mobile phase consisting of 20 mM NaH_2_PO_4 _(pH 3.5), and then centrifuged. The supernatant was analyzed using the RI-HPLC conditions described below. The reaction rate was obtained based on the relationship between reaction time and the concentrations of 5-FU and its metabolites 5-fluorodihydrouracil(5-FDHU), 2-fluoro-β-ureidopropionate (FUPA), and α-fluoro-β-alanine (FBAL). From this reaction rate and the protein concentration (determined separately), DPD activity (pmol/min per mg protein) was calculated.

#### HPLC conditions

The column was a YMC-Pack Pro C18 (AS-301-3, 4.6 × 100 min; YMC, Kyoto, Japan) kept at room temperature. The guard column was a Guard-Pak Puresil C (Waters, Milford, MA, USA). The mobile phase consisted of 20 mM phosphate buffer (pH 3.5) and the flow rate was 0.5 ml/min.

#### RI detection

The scintillation cocktail used was Pico Fluour40 (PerkinElmer Life Sciences, Boston, MA, USA). The scintillation flow rate was 3.0 ml/min, and the injection volume was 40 μl.

### OPRT activity

OPRT enzyme activity in samples of frozen tissue was measured by the paper disk method. The tissue sample was homogenized in 50 mM Tris-HCl (pH 7.5) containing 1.5 mM MgCl and 2 mM dithiothreitol. After ultracentrifugation (105000 × g, 1 h, 4°C), the supernatant was collected and incubated at 37°C with [^3^H]-5FU as the substrate. Aliquots of the reaction mixture were removed after 5, 10, and 15 min of incubation, and the reaction was stopped immediately by incubation in a 100°C water bath. After centrifugation, the supernatant was placed on an ion exchange filter paper made from diethylaminoethyl (DEAE)-cellulose, and washed to remove unreacted [^3^H]-5FU. The radioactivity of the [^3^H]-fluorouridine monophosphate (FUMP) formed was quantified to determine the concentration of FUMP. The reaction rate was obtained based on the relationship between the reaction time and the concentration of the FUMP formed. From this reaction rate and the protein concentration (determined separately), OPRT activity (pmol/min per mg protein) was calculated.

### Statistical Analysis

Results are presented as means ± SD. The relationship between disease severity (Dukes' B or C) and the activity of each enzyme was evaluated with the Mann-Whitney test. To evaluate the relationship between enzyme activity and survival period, the subjects were divided into high-activity and low-activity groups and differences were analyzed with the Pearson χ^2 ^statistic. Linear regression analysis between two variables was done using a simple regression method, the significance of which was quantified by analysis of variance (ANOVA). When disease-free survival was used as an end point, an event included recurrence of disease, death from cancer, and death from non-cancer causes. Survival curves were constructed by the Kaplan-Meier method. Multivariate analysis was performed using the Cox's proportional hazards regression model. A p value < 0.05 was considered significant.

## Results

### Relationship between enzyme activity and clinicopathological variables

Age, gender, and pathological findings such as histological type, site and depth of tumor and tumor size except for lymph node metastasis were similarly distributed between the two groups (Table 1). TS, DPD and OPRT activities are given in Table 2. In the tumor, mean values of TS, DPD and OPRT activities were 10.8 ± 8.0 pmol/min/mg protein, 28.1 ± 24.0 pmol/min/mg protein and 0.50 ± 0.11 nmol/min/mg-protein, respectively. The ranges of TS, DPD and OPRT activities in the tumor were 3.5–34.0 pmol/min/mg protein, 9.0–61.5 pmol/min/mg protein and 0.34–0.78 nmol/min/mg protein, respectively. No significant relationship was found between age or gender and activity of these enzymes. TS and DPD activities showed no clear relationship to any of the following pathological findings: histological type, lymph node metastasis, depth of tumor and tumor size. However, OPRT activity in the tumor was lower in Dukes' C than Dukes' B (0.41 ± 0.13 *vs*. 0.65 ± 0.11 pmol/min/mg protein, P < 0.01), while TS and DPD activities exhibited no clear relationship with tumor staging.

**Table 2:** Patient characteristics and enzymes activities

Factor	n	TS activity Tumor: Normal colon	DPDS activity Tumor: Normal colon	OPRT activity Tumor: Normal colon
Mean Age:	40	10.8 ± 8.0 2.1 ± 1.2	28.1 ± 24.0 29.9 ± 14.6	0.50 ± 0.11 0.23 ± 0.07
Age:				
young < 69	24	11.6 ± 8.7 2.6 ± 0.8	30.5 ± 28.9 28.6 ± 14.8	0.54 ± 0.10 0.20 ± 0.09
Old > 70	16	10.0 ± 8.1 1.6 ± 1.8	27.2 ± 20.7 30.7 ± 12.1	0.48 ± 0.14 0.25 ± 0.05
Gender:				
male	23	12.1 ± 9.8 2.2 ± 1.4	25.8 ± 27.7 28.8 ± 15.8	0.54 ± 0.13 0.20 ± 0.09
Female	17	9.9 ± 9.5 2.0 ± 1.1	32.3 ± 21.3 30.6 ± 11.2	0.49 ± 0.12 0.24 ± 0.07
Histological type				
Well	22	11.90 ± 5.6 2.3 ± 1.0	26.9 ± 21.8 31.8 ± 15.9	0.56 ± 0.12 0.20 ± 0.11
Moderately	18	10.0 ± 11.8 1.9 ± 1.5	30.5 ± 25.9 27.6 ± 11.8	0.45 ± 0.14 0.23 ± 0.05
Nodal status				
N(-)	22	9.5 ± 10.2 2.7 ± 1.8	27.9 ± 27.8 32.0 ± 12.1	0.65 ± 0.11* 0.22 ± 0.08
N(+)	18	12.5 ± 6.9 1.5 ± 0.8	30.0 ± 22.8 26.5 ± 17.2	0.41 ± 0.13* 0.23 ± 0.07
Tumor depth				
*s*.*s*.	26	10.0 ± 6.7 1.9 ± 0.9	27.5 ± 19.8 32.8 ± 11.5	0.54 ± 0.13 0.23 ± 0.08
*s*.*e*.	14	12.1 ± 9.8 2.3 ± 1.8	29.2 ± 29.7 26.1 ± 15.3	0.48 ± 0.11 0.23 ± 0.08
Tumor size				
> 5.0 cm	27	9.5 ± 6.6 2.6 ± 1.0	27.7 ± 27.8 35.7 ± 19.9	0.54 ± 0.13 0.22 ± 0.07
< 4.9 cm	13	12.5 ± 9.5 1.7 ± 1.9	30.9 ± 20.8 23.9 ± 9.9	0.48 ± 0.12 0.23 ± 0.09

*s*.*s*.: histological tumor invasion of subserosa, *s*.*e*.: histological tumor invasion of serosa

*P < 0.05 (p mol/min/mg-protein)

Values represent mean ± SD.

### Correlation between enzyme activity in colorectal tumor and adjacent normal tissue

**Figure 1:**
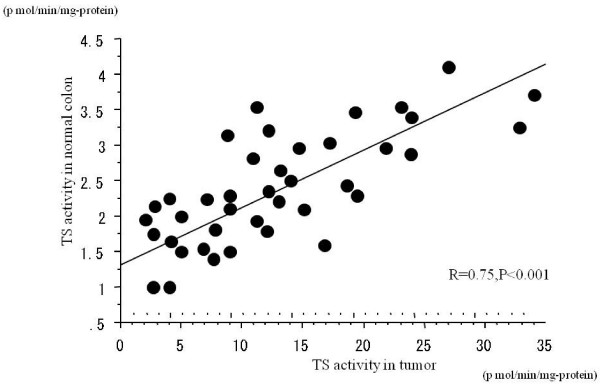
Correlation between TS activity in tumor and adjacent non-tumor tissue.

**Figure 2:**
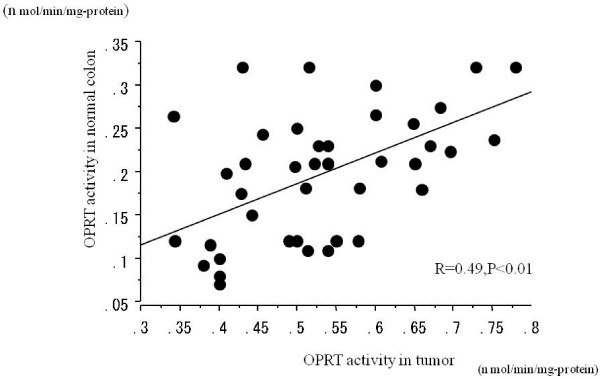
Correlation between OPRT activity in tumor and adjacent non-tumor tissue.

The mean activities of TS, DPD and OPRT in non-tumor sites were 2.1 ± 1.2 pmol/min/mg protein, 29.9 ± 14.6 pmol/min/mg protein and 0.23 ± 0.07 nmol/min/mg protein, respectively. The mean TS and OPRT activities were significantly higher (P < 0.01) in tumor tissue than in adjacent normal tissue, but no significant difference was found in DPD activity between tumor and adjacent normal tissue. The correlation between TS activity in tumor and adjacent normal tissue was high (r = 0.75, P < 0.001), while the correlation between OPRT activity in tumor and adjacent normal tissue was weaker although also statistically significant (r = 0.49, P < 0.01) (Fig. 1 and 2). No relationship existed between tumor/normal tissue ratio of enzyme activity and age, gender, pathological findings or Dukes' stage for any enzyme.

### Correlation of enzymes activity and prognosis

**Figure 3:**
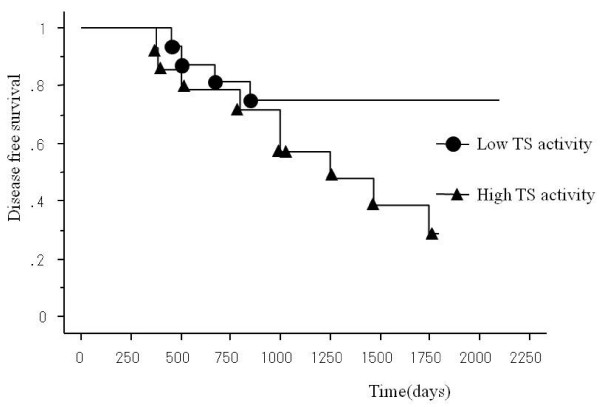
**Disease-free survival for patients with high TS activity and low TS activity.** The 5-year disease-free survival rates for high and low TS activity were 34% and 73%, respectively. The difference in survival was statistically significant. (P < 0.05)

**Figure 4:**
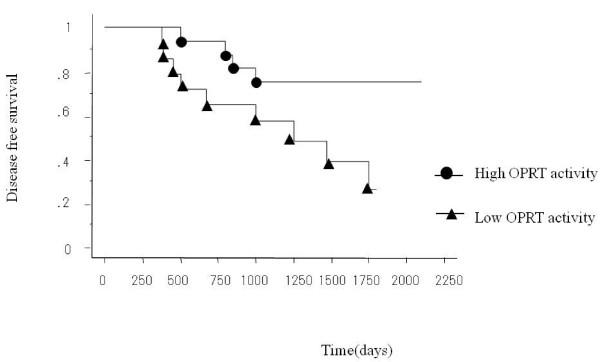
**Disease free survival for patients with high OPRT activity and low OPRT activity.** The 5-year disease-free survival rates for high and low OPRT activity were 75% and 28%, respectively. The difference in survival was statistically significant. (P < 0.05)

By the Kaplan-Meier method, there were no significant differences in disease-free survival between patients in Dukes' B and C stages, with the 5-year disease-free survival rates of 52% and 42% respectively. Disease-free survival was significantly better in the low TS activity group (TS < 10.7) than the high TS activity group (TS > 10.7) (P < 0.05) (Fig. 3) and also in the high OPRT activity group (OPRT > 0.51) than the low OPRT activity group (OPRT < 0.51) (P < 0.05), although no significant differences were found between high and low DPD groups (Fig. 4). The greatest increase in survival was observed for the group of patients exhibiting both low TS activity and high OPRT activity (78% in 5-year disease-free survival rate). Among patients with Dukes' B or Dukes' C, disease-free survival was significantly better in the high OPRT activity group and the low TS group, respectively. Nine variables (TS, DPD, OPRT, age, sex, tumor size, histological type, lymphatic metastasis and tumor depth) were analyzed using the Cox's proportional hazards regression model to determine the factors affecting the survival of colorectal cancer patients. Analyses showed TS activity (P = 0.05) and OPRT activity (P = 0.03) to be significant variables that independently predict postoperative survival (Table 3).

**Table 3:** Results of Cox proportional hazard regression analysis

Prognostic factors	P	Hazards ratio
Age (69 < vs. > 70)	0.28	0.18
Sex (male vs. female)	0.67	0.015
TS activity (high vs. low)	0.05	3.85
DPD activity (high vs. low)	0.90	0.06
OPRT activity (high vs. low)	0.03	5.04
Tumor size (< 4.9 vs. > 5.0 cm)	0.80	0.03
Lymphnode metastasis	0.15	1.36
(absent vs. present)		
Histology (well vs. mod)	0.90	0.01
Tumor depth (ss vs. se)	0.18	1.20

*s*.*s*.: histological tumor invasion of subserosa, *s*.*e*.:histological tumor invasion of serosa. Well: well differentiated adenocarcinoma, mod: moderately differentiated adenocarcinoma.

## Discussion

Here, we report that neither TS nor DPD activities significantly correlated with clinicopathological factors in patients undergoing radical resection for colon cancer. However, OPRT activity in tumors with lymph node metastasis was significant lower than in tumors without lymph node metastasis. This is similar to the observations of Ochiai et al., who showed that metastasis to the lymph nodes was associated with a significant reduction in the tumor/normal tissue ratio of ORPT activity[9]. The fact that the OPRT activity ratio for tumor tissue/normal tissue significantly decreases as tumor stage increases in colorectal carcinoma suggests that it may be possible to predict lymphatic metastasis by determining OPRT activity in tumor tissue prior to surgery. In contrast to our findings, Ochiai et al. also reported that DPD activity levels were high in poorly differentiated adenocarcinoma and mucinous carcinoma[9]. They also concluded that the expression of these enzymes may be associated with a poor prognosis for patients with poorly differentiated adenocarcinoma and mucinous carcinoma who have been treated with 5-FU.

Contradictory results have been reported in investigations of the relationship between these enzymes activities and clinicopathologic features. While we found no correlation between TS activity and tumor size, depth, staging or other pathological findings, Johnston et al. have reported that TS activity in tumor tissue was significantly correlated with tumor size, and that DPD activity in tumor tissue was significantly higher in the patients with liver metastasis than in those without metastasis[10]. Fujii et al.[8] reported that TS and OPRT activities were high in carcinomas with high proliferative activity. However, similar to our findings, they showed no significant differences in DPD activity with respect to clinicopathological variables.

The disparate conclusions reached regarding role of TS, DPD and OPRT in colorectal tumors may be explained by the different methodologies. The optimal method of assessing TS, DPD and OPRT expression is unclear, but current methods include immunohistochemistry (IHC), reverse transcriptase polymerase chain reaction (RT-PCR) and enzyme activity assay. The most common technique used for survival analysis is IHC, which qualitatively determines protein levels based on intensity of immunostaining. This method is difficult to standardize and cannot provide a measure of enzyme activity. RT-PCR allows for measuring marker expression in a highly sensitive manner but requires fresh samples and it too cannot provide a measure enzyme levels or activity. We chose to assay enzyme activity because this method not only provides an estimate of the absolute intracellular content, but also allows provides a measure enzyme functionality, although a disadvantage of this method is potential loss of enzyme activity due to protein instability.

In the few reports comparing the expression of enzymes involved 5-FU metabolism between tumor and normal tissue, expression of the initial 5-FU-anabolizing enzymes (OPRT, UP, TP, etc.) was elevated in human cancers as compared with normal tissues[9,11,12]. Our study similarly demonstrated higher TS and OPRT activities in tumors than adjacent normal mucosa although we did not note any significant difference in DPD activity between the two. These findings indicate that 5-FU metabolism is increased in tumor tissue and is regulated by both TS and OPRT[13]. Similar to our findings, Ichikawa[14] and Fujii[8] have also reported a correlation between low OPRT expression and worse prognoses or decreased responses to chemotherapy. The increased expression of these enzymes may promote proliferation of cancerous cells via increased pyrimidine nucleotide biosynthesis. In particular, biosynthesis of nucleic acid via increased salvage synthesis is thought to be closely connected to tumor growth. Using combined OPRT and TS quantitation, we are able to identify a subgroup of Dukes' B or C patients with a high recurrence rate and low disease-specific survival.

Our data also demonstrated that there was a significant correlation between the activities of TS and OPRT in tumor and disease-free survival (DFS) in patients receiving radical operation for colorectal carcinoma with adjuvant chemotherapy with UFT. An inverse correlation was observed between TS and DFS activities, while a positive relationship was seen between OPRT and DFS activities, as reported elsewhere [13-15]. Furthermore, we also found that patients with low TS activity and high OPRT activity survived longer, consistent with studies that have reported TS activity to be a good parameter by which to predict postoperative survival and to evaluate the effectiveness of 5-FU[16,17]. In a study by Edler et al., patients with low expression of TS in the tumor were found to have increased DFS compared to those with high TS activity[18]. Several groups have investigated the predictive value of DPD as an indication of sensitivity to 5 FU[19,20], suggesting that low levels of intratumoral DPD expression are associated with 5-FU responsiveness. However, our findings and the findings of Fujii et al. found no clear relationship between DPD activity and 5-FU sensitivity.[8] This discrepancy could be explained either by the methodological differences or the broad range in systemic DPD activity. Taking into account these considerations, the role of DPD an indicator of as tumor responsiveness for patients with DPD activity within the normal range awaits further clinical investigation in a prospective fashion before definitive conclusions.

## Conclusion

The activity levels of TS and OPRT in tumor tissue may be important prognostic factors for survival in Dukes' B and C colorectal cancers with radical operation and adjuvant chemotherapy with UFT. However, these conclusions are drawn from a limited retrospective study. Further prospective research that enlists patients with or without adjuvant chemotherapy will be required to establish the reliability of these parameters for clinical prediction of 5-FU sensitivity.

## Competing interests

The authors declare that they have no competing interests.

(Masashi ishikawa, M.D.PhD)

## Authors' contributions

Masashi Ishikawa carried out the molecular genetic studies, participated in the sequence alignment and drafted the manuscript. T Miyauchi participated in the design of the study and performed the statistical analysis. Y Kashiwagi conceived of the study, and participated in its design and coordination and helped to draft the manuscript.

## Pre-publication history

The pre-publication history for this paper can be accessed here:


